# Experiments on Chloride Binding and Its Release by Sulfates in Cementitious Materials

**DOI:** 10.3390/ma17143429

**Published:** 2024-07-11

**Authors:** Jian-Jun Dong, Yu-Xiao Zou, Xiao-Bao Zuo, Liang Li

**Affiliations:** School of Safety Science and Engineering, Nanjing University of Science & Technology, Nanjing 210094, China; dongjj@njust.edu.cn (J.-J.D.); xbzuo@sina.com (X.-B.Z.); liliang8531@163.com (L.L.)

**Keywords:** hardened cement paste, water-to-cement ratio, chloride binding, chloride release, sulfates

## Abstract

The aim of this study was to experimentally investigate the process of chloride binding and its sulfate-induced release in cementitious materials. The cementitious materials were replaced with hardened cement paste particles (HCPs) with water-to-cement ratios (w/c) of 0.35 and 0.45. A long-term immersion experiment of HCPs in 0.1 M sodium chloride solution was performed to investigate its chloride-binding capacity, and then it was immersed in sodium sulfate solutions with concentrations of 0.1 and 0.5 M to explore the release of chloride binding induced by sulfates. Silver nitrate titration and quantitative X-ray diffraction (QXRD) were used to measure the concentration of free chlorides in the solutions and the content of bound chlorides in HCPs, respectively. The results show that there is a higher chloride-binding capacity in HCPs with a w/c ratio of 0.45 compared to 0.35, and the content of chemically bound chlorides is associated with the formation and decomposition of Friedel’s and Kuzel’s salts in HCPs. The presence of sulfates can easily result in the release of bound chlorides in Friedel’s salt, but it cannot cause a complete release of bound chlorides in Kuzel’s salt. Physically bound chlorides are more easily released by sulfates than chemically bound chlorides, and a high w/c ratio or sulfate concentration can increase the release rate of bound chlorides in HCPs.

## 1. Introduction

Due to long-term exposure to marine, salt lake, and saline soil environments, concrete structures, such as bridges, roads, and harbors, are susceptible to the coupled attacks of chlorides and sulfates [1,2]. Chloride attack can cause the corrosion of steel reinforcements, and sulfate attack can deteriorate the mechanical properties of concrete, resulting in durability deterioration and lifetime reduction of concrete structures [3]. Chlorides from the environment can diffuse into concrete, and they are divided into bound chlorides in chloride-bearing phases and free chlorides in pore solution [4,5]. When the concentration of free chlorides exceeds the threshold at the steel reinforcement depth [6], it can cause the corrosion of steel reinforcements. According to the American Society of Civil Engineers (ASCE), approximately USD 76 billion is spent annually on the rehabilitation and retrofitting of concrete structures [7]. As the chloride-binding capacity (CBC) of cement hydration products increases, it effectively slows down the diffusion of free chlorides, thereby reducing the probability of steel reinforcement corrosion in concrete [8,9]. Lehner et al. [10] found that the appropriate content of supplementary cementitious materials such as zeolite, slag, and silica fume can decrease the diffusion coefficient of chlorides in concrete. However, environmental sulfates can diffuse into concrete and produce ettringite and gypsum, leading to the microstructure deterioration of concrete and eventually causing cracks [11]. Thus, the presence of sulfates can not only accelerate chloride diffusion but also influence the CBC of concrete due to their interactions [12]. Therefore, it is essential to investigate chloride binding and its release process induced by sulfates for evaluating the durability and lifetime of concrete structures in chloride–sulfate environments.

CBC can be characterized by the chemically and physically bound chlorides in concrete [13,14]. The mechanism of chemical binding is that free chlorides in the pore solution can react with cement hydration products to form chloride-containing AFm (hydrocalumite group) [15], such as Friedel’s salt [16] and Kuzel’s salt [17], which are known as chemically bound chlorides. Birnin-Yauri et al. [18] showed that the hydroxy-AFm (OH-AFm) can also react with free chlorides to form Friedel’s salt, and the CBC of OH-AFm was 126.5 mg/g when the concentration of free chlorides was above 0.015 M. In order to investigate the CBC of monosulfoaluminate (SO_4_-AFm) in concrete, Hirao et al. [19] put SO_4_-AFm into a 0.1 M sodium chloride solution to perform immersion experiments, and the results showed that its CBC was 6.22 mg/g. In addition, the experimental results from Florea et al. [20] also demonstrated that OH-AFm and SO_4_-AFm were the main phases of chemically bound chlorides in concrete, and their content was approximately 70% of the total bound chloride content. The physical binding of chlorides refers to the adsorption of the C-S-H gel surface in concrete [21,22], and these chlorides are called physically bound chlorides. Zibara et al. [23] pointed out that the physical binding capacity of C-S-H gel on free chlorides is mainly related to the Ca/Si molar ratio, and a higher Ca/Si ratio can form a higher physical binding capacity of C-S-H gel. Gou et al. [24] showed that the physical binding capacity of C-S-H gel increased and then decreased with the increase in the Ca/Si ratio, and the maximum physical binding capacity was achieved when the Ca/Si ratio of C-S-H gel reached 1.2. In general, the content of physically bound chlorides in C-S-H gel was approximately 25~28% of the total bound chlorides in concrete.

In chloride–sulfate environments, there is an obvious difference between chloride and sulfate diffusion in concrete, and chlorides can usually diffuse into concrete faster than sulfates [11,25]. First of all, diffused chlorides can be chemically and physically bound in concrete, and then they can be released by the latterly penetrated sulfates. This process demonstrates the initial binding of chlorides followed by their release due to sulfate exposure in concrete [26,27]. Recent studies have focused on the CBC of concrete in chloride environments and some isothermal adsorption curves [28,29,30], which are associated with the concentration of free chlorides and the content of bound chlorides. However, the time-dependent changes in the bound chloride content in concrete have been less frequently studied, and there is a lack of investigations into the release behavior of bound chlorides in concrete. In particular, the influence of sulfates and their concentration on the release process of bound chlorides in concrete has not been further studied.

Chloride binding and its release by sulfates in cementitious materials have a significant influence on the durability and lifetime of concrete structures in chloride–sulfate environments. To investigate chloride binding and its release process, hardened cement paste particles (HCPs) were used to replace the cementitious materials in order to perform a series of immersion experiments. Firstly, some HCPs were prepared and immersed in 0.1 M sodium chloride solution to investigate the chloride-binding process. Secondly, after the predetermined immersion time, they were removed from the sodium chloride solution, and then these chloride-containing HCPs were immersed in 0.1 and 0.5 M sodium sulfate solutions, respectively, to investigate the release process of bound chlorides. During the immersion experiments, the concentration of free chlorides in the solutions was determined by silver nitrate titration, while the content of bound chlorides in the HCPs was quantified through quantitative X-ray diffraction analysis. Based on the experimental data, the influence of the water-to-cement ratio and sulfates on the processes of chloride binding and sulfate-induced release in HCPs was investigated. These findings provide valuable insights into investigating chloride binding and its release process in cementitious materials in chloride–sulfate environments.

## 2. Materials and Methods

### 2.1. Raw Materials

**Figure 1:**
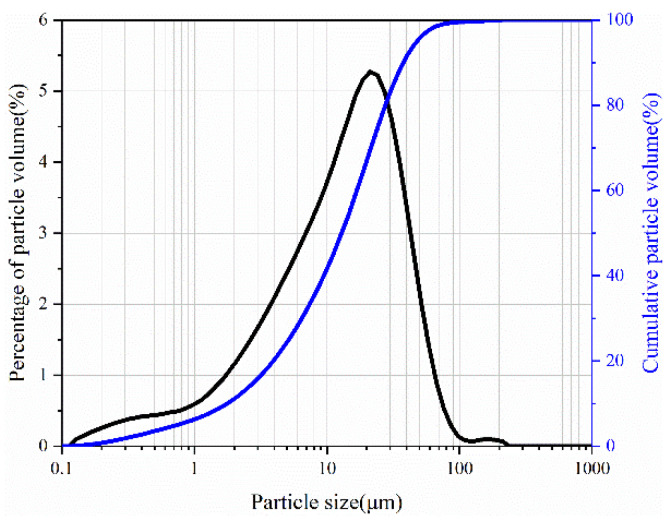
Particle size distribution of cement.

P·I 52.5 Portland cement complied with GB/T175 [31] was used in this study. The particle size distribution is illustrated in Figure 1. The chemical composition of cement was analyzed by X-ray fluorescence spectrometer (XRF), and its mineralogical composition was determined by X-ray diffraction (XRD). The chemical and mineralogical compositions are presented in Table 1. Deionized water was used for mixing the cement pastes.

### 2.2. Sample Preparation

Two hardened cement paste (HCP) specimens were prepared with water-to-cement ratios (w/c) of 0.35 and 0.45, as detailed in Table 2. The fresh cement pastes were cast into plastic molds measuring 40 mm × 40 mm × 160 mm and fully vibrated to ensure compaction. To prevent moisture evaporation, the exposed surfaces were covered with plastic wrap. After curing at ambient conditions for 24 h, the specimens were de-molded and subsequently cured for 120 days under standard conditions (20 ± 1 °C and relative humidity above 95 %) [32].

HCP specimens were crushed into particles sized 1.5 to 2.0 mm to ensure optimal contact with the immersion solution. These hardened cement paste particles (HCPs) were dried in a vacuum at 25 °C for 2 days and then stored in a desiccator filled with silica gel for immersion experiments.

### 2.3. Immersion Experiment

**Figure 2:**
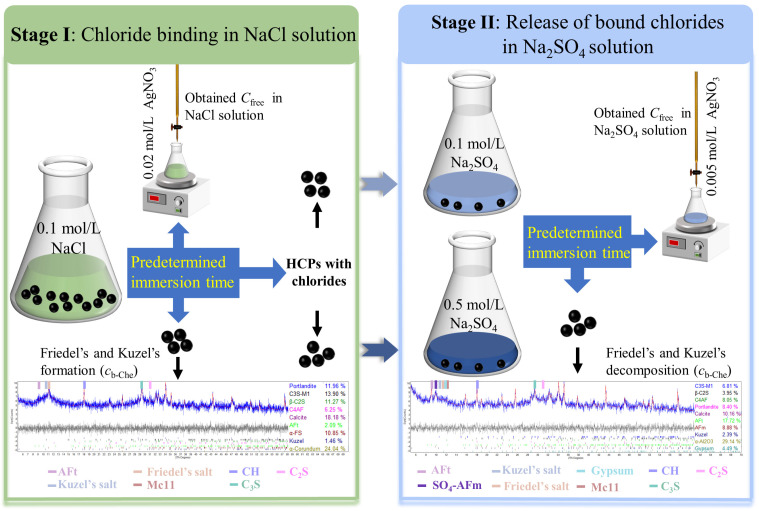
Schematic illustration of immersion experiment of HCPs in the solutions.

The process of initial chloride binding and subsequent release in HCPs can be achieved through long-term immersion experiments, first in a sodium chloride solution and then in a sodium sulfate solution. Thus, the immersion experiments were conducted in two stages (I, II), as shown in Figure 2.

Stage I: The prepared HCPs were immersed in conical flasks filled with 0.1 M sodium chloride solutions, maintaining a mass ratio of 1:10 between the particles and the solution. The flasks were sealed with cling film to prevent water evaporation, and the solution temperature was kept at 20 °C. After predetermined immersion times of 0.125, 0.25, 0.5, 2, 8, 32, and 128 days, the HCPs were extracted to measure the free chloride concentration ($C_{\mathrm{free}}$) in sodium chloride solutions using silver nitrate titration. The extracted HCPs were divided into three groups, one group was used to measure chemically bound chloride content ($C_{b\text{-}\mathrm{Che}}$), and the other two groups were reserved for Stage II immersion experiments. The bound chloride content in HCPs was quantitatively measured using quantitative X-ray diffraction (QXRD).

Stage II: The two remaining groups of HCPs from Stage I were immersed in 0.1 and 0.5 M sodium sulfate solutions, respectively, with immersion times identical to those in Stage I. After reaching the predetermined times, the HCPs were extracted from the sodium sulfate solutions. The free chloride concentration in sodium sulfate solutions was measured by silver nitrate titration, while the chemically bound chlorides in the HCPs were quantified using QXRD.

### 2.4. Measured Methods

#### 2.4.1. Silver Nitrate Titration

The concentrations of free chlorides in sodium chloride or sodium sulfate solutions were measured using silver nitrate titration [33], and its specific method is as follows. Firstly, 10 mL of the solution was removed from sodium chloride or sodium sulfate solutions, and then it was filtered and poured into a beaker. Secondly, two drops of 0.5% phenolphthalein were added to make it slightly red, and after it was neutralized with the dilute sulfuric acid, five drops of 5% potassium chromate solution were added to the beaker. Finally, the silver nitrate solution was gradually dropped into the beaker until the color of the solution changed from milky white to red, and its volume consumed at this time was recorded, so the concentration of free chlorides in the solutions could be calculated by

(1) $$C_{\mathrm{free}}=\frac{C_{{\mathrm{AgNO}}_{3}}V_{{\mathrm{AgNO}}_{3}}}{V}$$

where $C_{\mathrm{free}}$ is the concentration of free chlorides in sodium chloride or sodium sulfate solutions, M. $C_{{\mathrm{AgNO}}_{3}}$ is the concentration of silver nitrate solution, M. $V_{{\mathrm{AgNO}}_{3}}$ is the volume of silver nitrate solution consumed, L; V is the volume of sodium chloride or sodium sulfate solution, L.

#### 2.4.2. Quantitative X-ray Diffraction (QXRD)

The phase compositions in the HCPs at different immersion times in sodium chloride or sodium sulfate solution were analyzed by QXRD. The powder samples were prepared by mixing and finely grinding HCPs with anhydrous ethanol. To prevent the structural phase transformation of Friedel’s salt [34], the powder samples were placed in a vacuum drying oven at 30 °C. The dried samples were then mixed with 10 % α-Al_2_O_3_ (200 mesh, Shanghai Aladdin Bio-Chem Technology Co., LTD, Shanghai, China) by weight as the internal standard substance [35]. The XRD patterns were measured using a powder X-ray diffractometer (D8 Advance, Bruker, Ettlingen, Germany) with Cu-Kα radiation (λ = 0.154 nm, 40 kV, 40 mA) over a scanning range of 2θ = 5~70° at a step width of 2° per minute.

The XRD patterns of HCPs were characterized using the Inorganic Crystal Structure Database (ICSD). The phase compositions in HCPs were quantificationally analyzed by Rietveld refinement with TOPAS v4.2 software [36], which considered refined parameters such as scale factor, zero point, background coefficient, peak profile parameter, cell lattice parameter, phase shapes, and atomic coordinate. Additionally, since the cement used in this experiment contained no supplementary cementitious materials, the amorphous phase produced by cement hydration was considered to be C-S-H gel.

#### 2.4.3. Chloride Content

The total bound chloride content reflects the chloride-binding capacity of HCPs [37]. The bound chloride content in the HCPs after being immersed in sodium chloride solution can be expressed as

(2) $$C_{\mathrm{bound}}=\frac{1000M_{\mathrm{Cl}}\cdot V\cdot \left(C_{\mathrm{initial}}-C_{\mathrm{free},\mathrm{cl}}\right)}{m}$$

(3) $$C_{\mathrm{release}}=\frac{1000M_{\mathrm{Cl}}\cdot V\cdot C_{\mathrm{free},\mathrm{so}}}{m}$$

where $C_{\mathrm{bound}}$ and $C_{\mathrm{release}}$ are, respectively, the content of total bound and released chlorides in the HCPs, mg/g; $M_{\mathrm{Cl}}$ is the molar mass of chlorides, g/mol; m is the mass of HCPs, g; $C_{\mathrm{initial}}$ is the initial concentration of sodium chloride solution, M; $C_{\mathrm{free},\mathrm{cl}}$ and $C_{\mathrm{free},\mathrm{so}}$ are, respectively, the concentration of free chlorides in sodium chloride and sodium sulfate solutions, M.

The total bound chlorides in the HCPs can be divided into chemically and physically bound chlorides. The chemically bound chlorides in Friedel’s and Kuzel’s salts can be measured by QXRD. The ideal stoichiometry of Friedel’s and Kuzel’s salts is 3CaO·Al_2_O_3_·CaCl_2_·10H_2_O and 3CaO·Al_2_O_3_·0.5CaCl_2_·0.5CaSO_4_·10H_2_O, respectively. The content of physically bound chlorides can be regarded as the chlorides bound by C-S-H gel. Thus, the content of physically bound chlorides can be calculated by subtracting the chemically bound chloride content from the total bound chloride content [38]. The content of total released chlorides in the HCPs can be measured using silver nitrate titration during the immersion experiment in Stage II, and the content of total released chlorides in HCPs can be obtained from the concentration of free chlorides in sodium sulfate solution. It should be noted that the release of the bound chlorides caused by sulfates in HCPs can be divided into chemically and physically released chlorides. The content of chemically released chlorides can be determined by the chlorides released from the decomposition of Friedel’s and Kuzel’s salts, which can be measured by QXRD. Therefore, the content of physically released chlorides is then calculated by subtracting the chemically released chloride content from the total released chloride content.

## 3. Results and Discussion

### 3.1. Chloride-Binding Capacity

#### 3.1.1. Chloride-Binding Process

**Figure 3:**
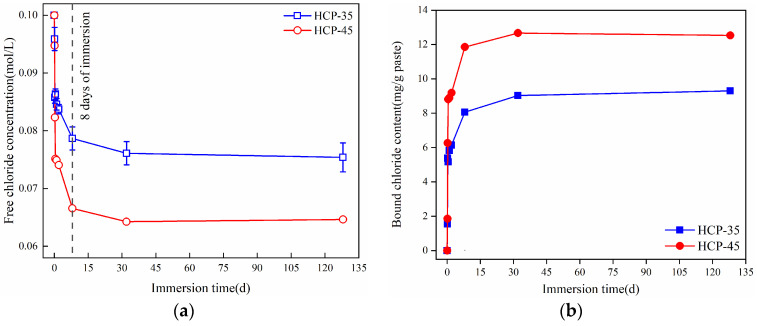
Changes of free chloride concentration and bound chloride content during immersion in sodium chloride solution. (**a**) Free chloride concentration. (**b**) Bound chloride content.

Figure 3 shows the changes in free chloride concentration in sodium chloride solution and bound chloride content in hardened cement paste particles (HCPs) with the immersion time. In Figure 3a, it is evident that the changes in free chloride concentration exhibit two stages during the immersion in sodium chloride solution. During the initial stage (0~8 d), both HCP-35 and HCP-45 show a significant decrease in free chloride concentrations as immersion time increases. Notably, HCP-45 shows a higher rate of decrease compared to HCP-35, indicating a higher bound chloride content in HCP-45 than in HCP-35. For instance, at 8 days of immersion, the concentrations of free chlorides in HCP-35 and HCP-45 were reduced to 21.0 and 33.0% of their initial concentrations in sodium chloride solution, respectively. During the second stage of immersion (8~128 days), the decrease in free chloride concentrations slows down compared to the initial stage. By 128 days of immersion, the free chloride concentration in HCP-35 (0.075 M) and HCP-45 (0.065 M) was decreased by 5.1 and 3.0%, respectively, compared to the concentration of free chlorides at 8 days of immersion. These results highlight that the higher water-to-cement ratios (w/c) of the HCPs enable them to bind more free chlorides, leading to the observed differences in chloride binding capacity between HCP-35 and HCP-45 during the immersion experiments.

To further investigate the influence of w/c on the chloride-binding capacity (CBC) of HCPs, the content of bound chlorides in the HCPs was calculated using Equation (2), and their changes with the immersion time are presented in Figure 3b. It can be seen from the figure that the content of bound chlorides in HCP-45 was higher than that in HCP-35, and both of them exhibited a rapid increase in bound chloride content during the initial 8 days of immersion. By 8 days of immersion, the content of bound chlorides in HCP-35 and HCP-45 has stabilized at values approximately 1.51 and 1.36 times higher than those at 2 days of immersion, respectively. This behavior can be attributed to the complete reaction of phases such as C-S-H and AFm with free chlorides within the initial 8 days of immersion, reaching chloride saturation levels. Previous studies have reported similar results [39], supporting this observation. Therefore, the content of bound chlorides in HCPs at 128 days of immersion in sodium chloride solution can be regarded as their CBC. At this point, the CBC in HCP-35 and HCP-45 were 9.30 and 12.53 mg/g, respectively. Obviously, increasing the w/c can improve the CBC of HCPs.

#### 3.1.2. Phase Composition

**Figure 4:**
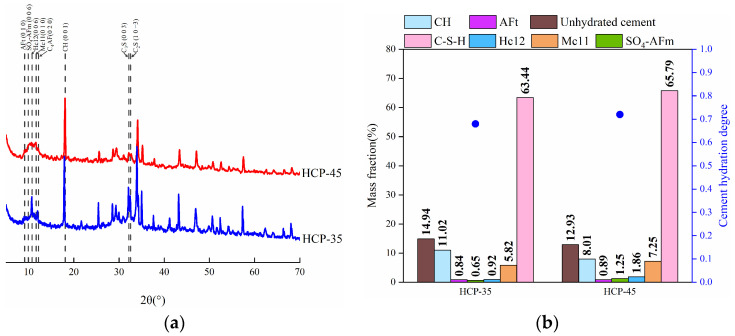
XRD patterns, phase compositions, and cement hydration degree of HCPs. (**a**) XRD patterns. (**b**) Phase compositions and cement hydration degree.

As reported in [40], AFm phases and C-S-H gel play significant roles in the CBC of HCPs. In this study, the XRD analysis was performed to determine the phase compositions and cement hydration degree of the HCPs, and the results are presented in Figure 4. The cement hydration degree was calculated as the weighted average of the hydration degrees of the four main clinker phases: C_3_S, C_2_S, C_3_A, and C_4_AF. The hydration degree of each phase was determined by comparing its content before and after hydration. The content in unhydrated cement was considered as the total amount, and the remaining content after hydration was taken as the unhydrated amount. The calculation method can be expressed as

(4) $$DoH=\frac{\sum_{1}^{i}w_{i}\cdot DoH_{i}}{\sum_{1}^{i}w_{i}}\;\left(i=C_{3}S,C_{2}S,C_{3}A,C_{4}AF\right)$$

where DoH is the cement hydration degree; $w_{i}$ is the initial mass fraction of *C*_3_*S*, *C*_2_*S*, *C*_3_*A*, and *C*_4_*AF* in unhydrated cement, respectively; $DoH_{i}$ is the hydration degree of *C*_3_*S*, *C*_2_*S*, *C*_3_*A*, and *C*_4_*AF* in HCP specimen, respectively.

It can be seen from the figure that, the contents of AFm phases, such as SO_4_-AFm, hemicarboaluminate (Hc12), and monocarboaluminate (Mc11), were higher in HCP-45 compared to HCP-35. Conversely, the content of calcium hydroxide (CH) was lower in HCP-45 (8.01%) than in HCP-35 (11.02%), attributed to CH consumption during AFm phase formation in the hydration process [41]. HCP-45 also exhibited a higher cement hydration degree (0.72) than HCP-35 (0.68), suggesting that a greater hydration degree can enhance the CBC of HCPs. It is important to note that the HCP specimens were cured under standard curing conditions (as described in Section 2.2) for 120 days, not submerged in water. This curing method, while providing high humidity, may result in a lower cement hydration degree compared to underwater curing, which could explain the observed hydration values. In addition, both HCP-35 and HCP-45 showed a relatively high content of C-S-H, and the C-S-H content in HCP-35 and HCP-45 was 63.44 and 72.37%, respectively. Thus, a high w/c can increase the content of AFm phases and C-S-H in HCPs.

**Figure 5:**
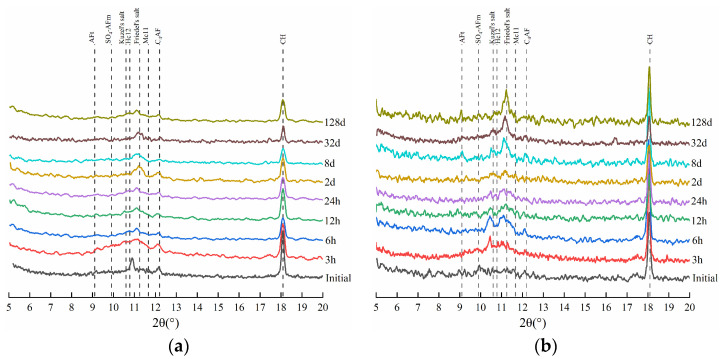
XRD patterns of HCPs during the sodium chloride immersion. (**a**) HCP-35. (**b**) HCP-45.

To further investigate the evolution of phase compositions in the HCPs in sodium chloride solution, Figure 5 presents the XRD patterns of HCP-35 and HCP-45 at different immersion times. It can be seen from the figure that, the intensity of the diffraction peak of CH shows a significant decrease with the immersion time, indicating that CH can influence the process of chloride binding in the HCPs. At the initial 32 days of immersion, the diffraction peak of SO_4_-AFm gradually disappeared, while the intensity of the diffraction peaks corresponding to Friedel’s and Kuzel’s salts increased. This observation indicates the involvement of SO_4_-AFm in the formation of Friedel’s and Kuzel’s salts, with a change in the bound chloride contents of these salts reflecting the chemical binding capacity of chlorides in the HCPs. In addition, during the sodium chloride immersion, the diffraction peak of ettringite (AFt) remains unchanged, indicating that AFt cannot participate in the chloride-binding process and maintains its chemical stability in sodium chloride solution.

#### 3.1.3. Binding of Free Chlorides

**Figure 6:**
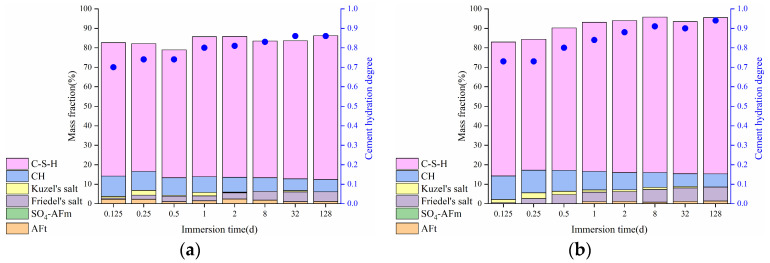
Changes of phase compositions in HCPs during the immersion in sodium chloride solution. (**a**) HCP-35; (**b**) HCP-45.

The change of phase compositions in the HCPs is presented in Figure 6, where the cement hydration degree is also marked. As observed from the figure, the cement hydration degree has an increase with immersion time, leading to the formation of additional hydration products that contribute to higher bound chloride content in the HCPs. During the initial 32 days of immersion, the content of Friedel’s salt in the HCPs shows a steady increase. However, after 32 days of immersion, there is no significant change in Friedel’s salt content, indicating saturation in its formation in the HCPs. Additionally, both Friedel’s and Kuzel’s salts were formed in the HCPs during sodium chloride immersion, with their content reaching a maximum value at 6 h of immersion. At this point, the content of Kuzel’s salt in HCP-35 and HCP-45 were 2.42 and 2.93%, respectively, while no Kuzel’s salt can be observed in the XRD patterns of HCP-35 and HCP-45 at 128 days of immersion, demonstrating poor chemical stability of Kuzel’s salt during prolonged exposure to sodium chloride solution.

**Figure 7:**
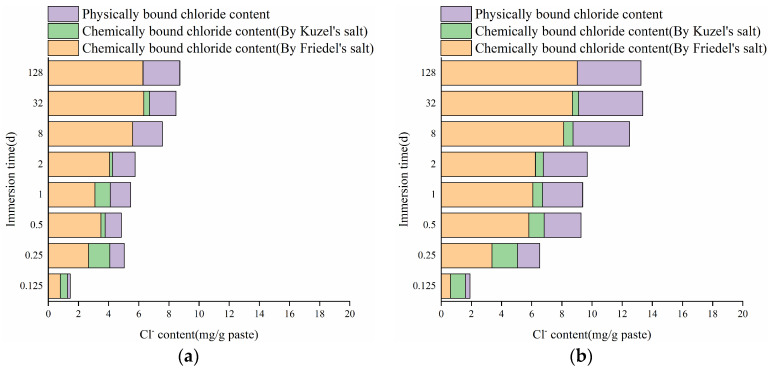
Changes of chemically and physically bound chloride contents in HCPs. (**a**) HCP-35; (**b**) HCP-45.

According to Figure 3b and Figure 6, the content of physically bound chlorides can be determined by subtracting the chemically bound chlorides from the total bound chlorides. Figure 7 presents the changes in chemically and physically bound chloride contents in the HCPs with immersion time. It can be observed from the figure that the chemically bound chlorides were mainly related to the generation of Friedel’s salt, and its content increased steadily during immersion. At the initial 6 h of immersion, the total bound chloride content in HCP-45 surpasses that in HCP-35, demonstrating that a higher w/c results in a higher CBC at the initial stage of immersion. At 12 h of immersion, the content of chemically bound chlorides in HCP-45 was 5.81 mg/g, whereas HCP-35 requires 8 days to achieve a similar content of 5.60 mg/g. Similarly, the content of physically bound chlorides also increased with immersion time, and a high w/c of HCPs has also a good physical binding capacity of chlorides. At 128 days of immersion, the contents of physically bound chlorides in HCP-45 and HCP-35 were 4.23 and 2.44 mg/g, respectively, and C-S-H contents in HCP-35 and HCP-45 were 73.84 and 80.22%, respectively. Based on the results from Zhang et al. [42], a higher w/c can result in the generation of more C-S-H with a high Ca/Si ratio in the HCPs. This higher Ca/Si ratio of C-S-H can enhance its capacity for physical binding [43]. Furthermore, a higher w/c typically results in higher porosity of the HCP specimen, thereby increasing the surface area available for chloride interaction. Therefore, the higher content of physically bound chlorides in HCP-45 compared to HCP-35 can be attributed to two main factors: increased porosity and surface area available for chloride interaction, and the potential formation of C-S-H with a higher Ca/Si ratio, which enhances its physical binding capacity of chlorides.

Based on the fitting of the data presented in Figure 7, the contents of chemically and physically bound chlorides in the HCPs can be obtained. Their variation with immersion time can be expressed as

(5) $$C_{b\text{-}\mathrm{Che}}=\frac{\left(29.5w_{c}-4.4\right)t}{0.26+t}$$

(6) $$C_{b\text{-}\mathrm{Phy}}=\frac{\left(18.4w_{c}-4.5\right)t}{0.26+t}$$

where $C_{b\text{-}\mathrm{Che}}$ and $C_{b\text{-}\mathrm{Phy}}$ are, respectively, the contents of chemically and physically bound chlorides, mg/g; $w_{c}$ is the w/c of HCPs; *t* is the immersion time, d.

By differentiating Equations (5) and (6) with respect to the immersion time *t*, the chemical and physical binding rates of chlorides in the HCPs can be obtained as follows

(7) $$v_{b\text{-}\mathrm{Che}\text{-}w_{c}}=\frac{7.67w_{c}-1.14}{{\left(0.26+t\right)}^{2}}$$

(8) $$v_{b\text{-}\mathrm{Phy}\text{-}w_{c}}=\frac{4.78w_{c}-1.17}{{\left(0.26+t\right)}^{2}}$$

where $v_{b\text{-}\mathrm{Che}\text{-}w_{c}}$ and $v_{b\text{-}\mathrm{Phy}\text{-}w_{c}}$ are, respectively, the chemical and physical binding rates of chlorides in HCPs with different w/c, mg/(g·d).

**Figure 8:**
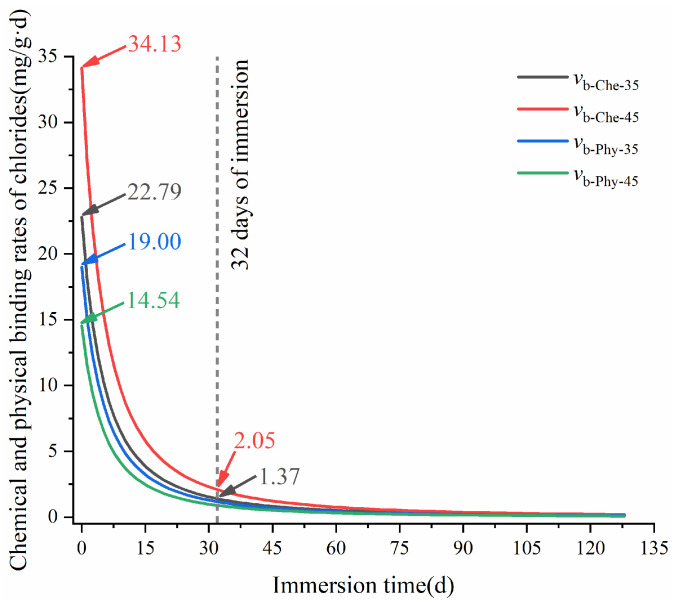
Changes in chemical and physical release rates of chlorides in HCPs during the immersion in sodium chloride solution.

Based on Equations (7) and (8), the chemical and physical binding rates of chlorides in HCP-35 and HCP-45 with immersion time are presented in Figure 8. As observed in the figure, the chemical and physical binding rates of chlorides in the CPs decreased with immersion time, and the relationship among the chemical and physical binding rates of chlorides in the HCPs was $v_{b\text{-}\mathrm{Che}\text{-}45}$ > $v_{b\text{-}\mathrm{Che}\text{-}35}$ > $v_{b\text{-}\mathrm{Phy}\text{-}45}$ > $v_{b\text{-}\mathrm{Phy}\text{-}35}$, which demonstrates that the CBC of HCPs was mainly associated with its chemical binding capacity. During the initial 32 days of immersion, both chemical and physical binding rates of chlorides exhibit a rapid decrease. For instance, at 32 days of immersion, the chemical binding rates of chlorides in HCP-35 (1.37 mg/g·d) and HCP-45 (2.05 mg/g·d) were, respectively, 1.20 and 2.39 times higher than their physical binding rates. This observation highlights that a high w/c is advantageous for improving the chemical binding rate of chlorides in the HCPs.

### 3.2. Sulfate-Induced Release of Bound Chlorides

#### 3.2.1. Release Process of Bound Chlorides

**Figure 9:**
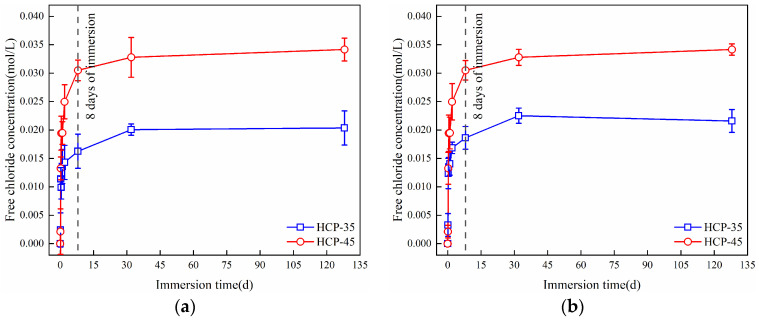
Changes of free chloride concentration during the immersion in sodium sulfate solution. (**a**) 0.1 M sodium sulfate; (**b**) 0.5 M sodium sulfate.

After HCPs immersed in sodium sulfate solution, their bound chlorides can be released by sulfates, and this can result in a reduction of total bound chloride content in the HCPs, which can increase the concentration of free chlorides in sodium sulfate solutions. The changes in free chloride concentration in 0.1 and 0.5 M sodium sulfate solutions with immersion time are presented in Figure 9. It can be seen from the figure that, the concentration of free chlorides in sodium sulfate solutions increased with immersion time, with higher concentrations observed in HCP-45 compared to HCP-35 at the same immersion time, reflecting the higher bound chloride content in HCP-45. The concentration of free chlorides showed a significant increase during the initial 8 days of immersion. Subsequently, the free chloride concentrations gradually increased until they stabilized, and there was basically no change in the concentrations of free chlorides in both 0.1 and 0.5 M sodium sulfate solutions.

#### 3.2.2. Phase Composition

**Figure 10:**
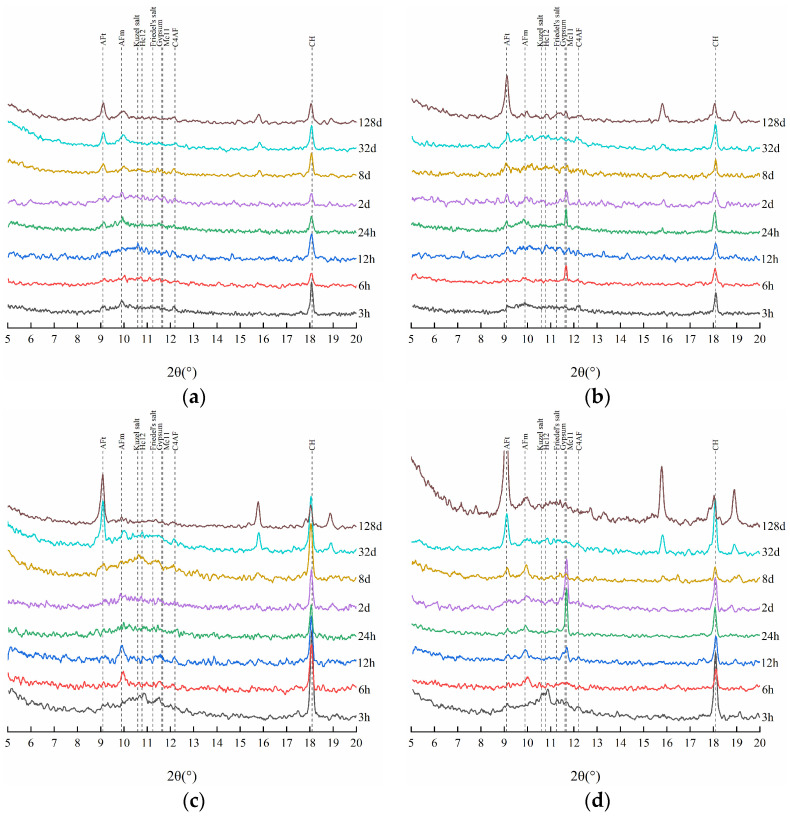
XRD patterns of HCPs during the immersion in sodium sulfate solution. (**a**) HCP-35 in 0.1 M sodium sulfate solution; (**b**) HCP-35 in 0.5 M sodium sulfate solution; (**c**) HCP-45 in 0.1 M sodium sulfate solution; (**d**) HCP-45 in 0.5 M sodium sulfate solution.

**Figure 11:**
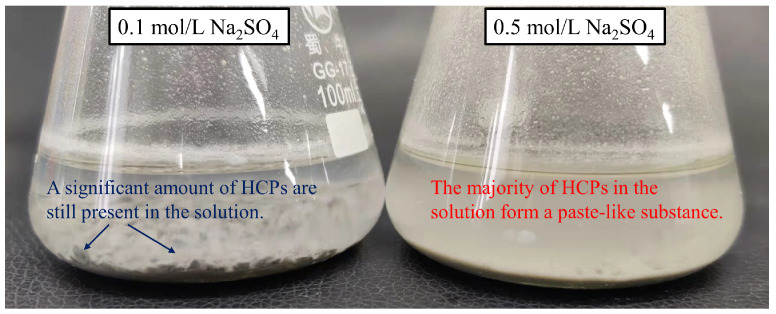
Photograph of HCP-45 after 128 days of immersion.

In order to analyze the evolution of phase compositions in the HCPs during sodium sulfate solution immersion, the XRD patterns of HCPs after different immersion times are presented in Figure 10. It can be seen from the figure that the diffraction peaks of Friedel’s and Kuzel’s salts decrease with immersion time, indicating that sulfates can cause the decomposition and the release of chemically bound chlorides in these salts. After the disappearance of these peaks, a maximum of free chloride concentration in sodium sulfate solution occurs. Additionally, the intensity of the AFt diffraction peak is higher in 0.5 M sodium sulfate solution compared to 0.1 M sodium sulfate solution, and a clear diffraction peak of gypsum can be seen in the XRD patterns, indicating that there is a sulfate attack in the HCPs [44]. Figure 11 further illustrates that sulfate attack is more pronounced in HCP-45 than in HCP-35, as evidenced by the swelling and cracking of the specimens [45]. This increased damage in HCP-45 is attributed to its higher content of AFm phases and C-S-H compared to HCP-35.

#### 3.2.3. Release of Bound Chlorides

**Figure 12:**
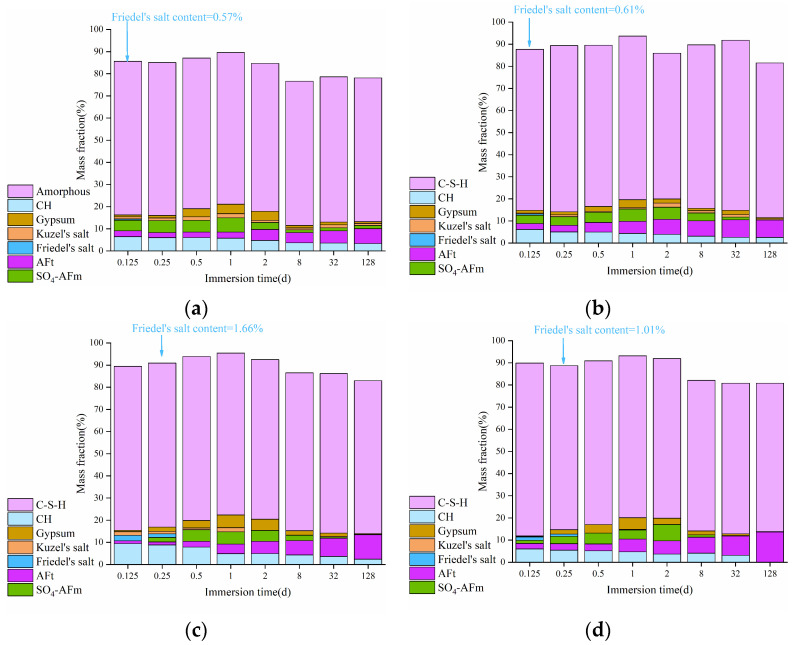
Changes of phase compositions in HCPs during the immersion in sodium sulfate solution. (**a**) HCP-35 in 0.1 M sodium sulfate solution; (**b**) HCP-35 in 0.5 M sodium sulfate solution; (**c**) HCP-45 in 0.1 M sodium sulfate solution; (**d**) HCP-45 in 0.5 M sodium sulfate solution.

Figure 12 illustrates the changes in phase compositions in the HCPs during sodium sulfate immersion. It can be observed from the figure that the content of Friedel’s salt in the HCPs decreases with immersion time, and Friedel’s salt in HCP-35 completely disappears after 6 h of immersion. In contrast, the content of Friedel’s salt in HCP-45 is still 1.01% after 12 h of immersion in 0.5 M sodium sulfate solution, indicating that a high w/c can slow down the release of chemically bound chlorides in the HCPs. Moreover, the content of SO_4_-AFm initially increased and then decreased with immersion time, while the content of AFt gradually increased. This trend indicates that SO_4_-AFm acts as an intermediate product in the transformation from Friedel’s salt to AFt. However, the content of Kuzel’s salt in the HCPs shows no apparent change with immersion time, indicating that the chemically bound chlorides in Kuzel’s salt are difficult to release by sulfates.

**Figure 13:**
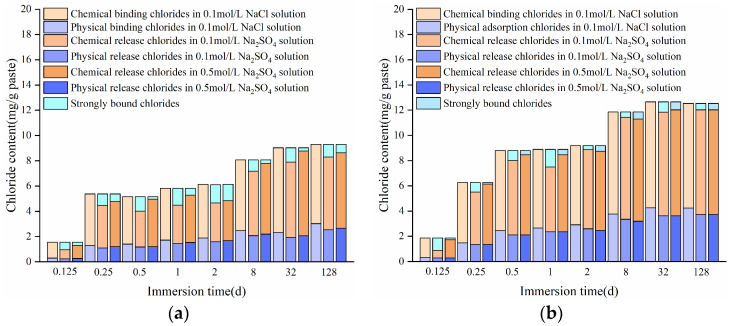
Changes of chloride contents in HCPs during the immersion in sodium chloride and sodium sulfate solutions. (**a**) HCP-35; (**b**) HCP-45.

Based on the findings in Figure 6, the changes in bound chloride contents in the HCPs after sodium chloride immersion are shown in Figure 13. As seen from the figure, the proportion of chemically bound chlorides exceeded 70% of the total bound chlorides in the HCPs during sodium chloride immersion. The changes in the w/c of HCPs have minimal influence on the content of strongly bound chlorides, which cannot be released by sulfates. Obviously, both chemically and physically bound chlorides in the HCPs resist complete release by sulfates. Specifically, in 0.1 and 0.5 M sodium sulfate solutions, the average contents of chemically bound chlorides were 82 and 89.9% in HCP-35, and 85 and 91% in HCP-45, respectively. Furthermore, the content of physically released chlorides exceeded chemically released chlorides in the HCPs, indicating that physically bound chlorides were more readily released by sulfates. This result is attributed to the weaker bonding force between C-S-H and physically bound chlorides compared to the strong chemical bonds between AFm phases and chemically bound chlorides, as reported by Wang et al. [46].

Based on the results shown in Figure 13, the change of chemically and physically released chloride contents in the HCPs with immersion time can be expressed by

(9) $$C_{r\text{-}\mathrm{Che}}=\frac{\left(35.1w_{c}+0.9c_{\mathrm{sul}}-6.78\right)t}{0.46+t}$$

(10) $$C_{r\text{-}\mathrm{Phy}}=\frac{\left(18.25w_{c}+0.11c_{\mathrm{sul}}-4.66\right)t}{0.46+t}$$

where $C_{r\text{-}\mathrm{Che}}$ and $C_{r\text{-}\mathrm{Phy}}$ are, respectively, the contents of chemically and physically released chlorides, mg/g; $c_{\mathrm{sul}}$ is the concentration of sulfates in solution, M.

Similarly, after sodium sulfate immersion experiments, the chemical and physical release rates of bound chlorides in the HCPs can be obtained by differentiating Equations (9) and (10) with respect to the immersion time *t*, and they are expressed by

(11) $$v_{r\text{-}\mathrm{Che}\text{-}c_{\mathrm{sul}}\text{-}w_{c}}=\frac{16.15w_{c}+0.41c_{\mathrm{sul}}-3.12}{{\left(0.46+t\right)}^{2}}$$

(12) $$v_{r\text{-}\mathrm{Phy}\text{-}c_{\mathrm{sul}}\text{-}w_{c}}=\frac{8.40w_{c}+0.05c_{\mathrm{sul}}-2.14}{{\left(0.46+t\right)}^{2}}$$

where $v_{r\text{-}\mathrm{Che}\text{-}c_{\mathrm{sul}}\text{-}w_{c}}$ and $v_{r\text{-}\mathrm{Phy}\text{-}c_{\mathrm{sul}}\text{-}w_{c}}$ are, respectively, the chemical and physical release rates of bound chlorides in HCPs with different w/c after immersed in $c_{\mathrm{sul}}$ M sulfate solution, mg/(g·d).

**Figure 14:**
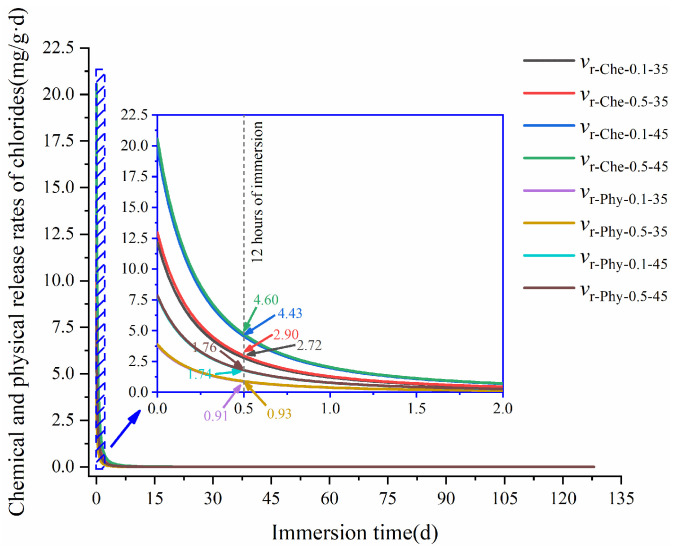
Changes of chemical and physical release rates of bound chlorides in HCPs during the immersion in sodium sulfate solution.

According to Equations (11) and (12), Figure 14 shows the changes in chemical and physical release rates of bound chlorides in the HCPs with immersion time. It can be seen from the figure that both chemical and physical release rates of bound chlorides in HCPs decreased with immersion time. During the initial 6 h of immersion, the relationship among the chemical and physical release rates in the HCPs were $v_{r\text{-}\mathrm{Che}\text{-}0.5\text{-}45}$ > $v_{r\text{-}\mathrm{Che}\text{-}0.1\text{-}45}$ > $v_{r\text{-}\mathrm{Che}\text{-}0.5\text{-}35}$ > $v_{r\text{-}\mathrm{Che}\text{-}0.1\text{-}35}$ > $v_{r\text{-}\mathrm{Phy}\text{-}0.5\text{-}45}$ > $v_{r\text{-}\mathrm{Phy}\text{-}0.1\text{-}45}$ > $v_{r\text{-}\mathrm{Phy}\text{-}0.5\text{-}35}$ > $v_{r\text{-}\mathrm{Phy}\text{-}0.1\text{-}35}$, which indicates that the release of bound chlorides in the HCPs was mainly associated with its chemical release rates. At the initial 12 h of immersion, a high w/c can increase the chemical and physical release rates of bound chlorides in the HCPs. In addition, a high concentration of sulfate can also increase the chemical and physical release rates of bound chlorides in the HCPs. This is because a high concentration of sulfates can increase its interaction with bound chlorides. Furthermore, sulfate-induced volume expansion further facilitated the release of bound chlorides from HCPs.

## 4. Conclusions

In this paper, the process of binding and sulfate-induced release of chlorides in cementitious materials, which were replaced by hardened cement paste particles (HCPs), have been investigated through long-term immersion experiments in sodium chloride and sodium sulfate solutions. The time-dependent changes in free chloride concentration in the solutions and bound chloride content in the HCPs with different water-to-cement ratios (w/c) were analyzed. Some conclusions can be drawn, as follows:

1. The content of chemically bound chlorides in the HCPs accounts for more than 70% of the total bound chloride content. This is mainly determined by the content of chlorides bound in Friedel’s salt. HPCs with a high w/c exhibit better chloride-binding capacity (CBC) than those with a low w/c.
2. A high concentration of sulfates can accelerate the release of bound chlorides in the HCPs. The chemically bound chlorides can be released due to the sulfate-induced decomposition of Friedel’s and Kuzel’s salts, and the bound chlorides in Kuzel’s salt are not easier to release by sulfates compared to those in Friedel’s salt.
3. Both the w/c and sulfate concentration significantly influence the chloride-binding process in cementitious materials, and a high w/c results in high binding rates of chlorides, while increased w/c and sulfate concentration can increase the release rates of chlorides.
4. The influence of sulfates on the CBC of HCPs in cementitious materials has been investigated, and it can be quantitatively characterized by the contents of chemically or physically bound chlorides and their release by sulfates. The results of this study provide insights into the interaction between chlorides and sulfates, and further model the coupled chloride and sulfate diffusion in concrete and structures exposed to chloride–sulfate environments.

## Figures and Tables

**Table 1:** Chemical and mineralogical compositions of cement (wt. %).

Chemical compositions	CaO	SiO_2_	Fe_2_O_3_	Al_2_O_3_	MgO	K_2_O	TiO_2_	Na_2_O	P_2_O_5_	SO_3_
	60.65	19.58	3.8	7.17	4.03	1.09	0.34	0.31	0.06	2.55
Mineralogical compositions	C_3_S	C_2_S	C_4_AF	C_3_A	Gypsum	Calcite	Periclase	Anhydrite	Lime	Amorphous
	40.60	20.37	13.37	8.64	2.46	3.77	4.71	1.36	0.52	4.21

**Table 2:** Mix proportion of HCP specimens.

Sample	Cement (wt. %)	Water (wt. %)	w/c
HCP-35	74.07	25.93	0.35
HCP-45	68.97	31.03	0.45

## Data Availability

Data are contained within the article.
